# Framing health taxes: a scoping review

**DOI:** 10.1136/bmjgh-2023-012055

**Published:** 2023-10-09

**Authors:** Adam D Koon, Robert Marten

**Affiliations:** 1 Department of International Health, Johns Hopkins Bloomberg School of Public Health, Baltimore, Maryland, USA; 2 Secretariat, WHO Alliance for Health Policy and Systems Research, Geneva, Switzerland

**Keywords:** Review, Health policy, Health systems

## Abstract

Health taxes are increasingly positioned as effective policy instruments for curbing non-communicable disease, improving health and raising government revenues. Their allure has caused many health advocates to look beyond tobacco and alcohol to other harmful products such as sugar-sweetened beverages (SSBs), salty foods, fatty foods and fossil fuels. These efforts, however, directly conflict with commercial actors’ interests. Both pro-tax health advocates and anti-tax industry representatives seek to frame health tax policy in favourable ways. Yet, little is known about which types of frames resonate in which settings, or how they deploy morals and values in their attempts to persuade. To fill this gap, we conducted a scoping review on framing health taxes using six databases in 2022. A total of 40 peer-reviewed empirical research articles, from 2006 to 2022, were identified from 20 different countries. Most research was conducted in high-income countries, published in the last 4 years and increasingly focused on excise taxes for SSBs. Studies captured multiple actors constructing context-specific frames, often tied to broader economic, health and administrative considerations. Actors also engaged in a range of political activities in addition to framing. We found some evidence that anti-tax framing strategies potentially incorporated a broader array of morals and social values. More in-country comparative research, particularly from low/middle-income countries, is needed to understand the politics of framing health taxes. We argue that these insights can improve efforts to advance health taxes by constraining corporate power, improving population level health and promoting greater social harmony.

**WHAT IS ALREADY KNOWN ON THIS TOPIC**

- Health taxes have been shown to reduce the burden of non-communicable diseases and raise revenue around the world.

**WHAT THIS STUDY ADDS**

- This research shows that evidence is rapidly increasing, especially from high-income countries and for sugar-sweetened beverage taxes, about how to frame health taxes. This includes developing grassroots arguments that resonate with local constituents, launching intensive media campaigns, reassuring voters that revenue generated from health taxes funds social programs, making greater use of health professional associations in health tax debates and developing different kinds of frames that tap a broader array of morals and social values (instead of searching for a single strong frame).

**HOW THIS STUDY MIGHT AFFECT RESEARCH, PRACTICE OR POLICY**

- This research will help health advocates think strategically about ways to frame different features of health tax policy in order to generate support from lawmakers and the general public.

## Introduction

Health taxes ignite debate. They are both new and old, simple and complex, effective and flawed. On the surface, consumer preferences are personal, and overt efforts to change them are inherently uncomfortable. Recognising this, we endorse the semantic shift from the stigmatising ‘sin taxes’ to ‘health taxes’ in labelling fiscal measures to control health-harming commodities (eg, alcohol, tobacco, sugar, salt, fossil fuels among potential others). But health taxes are also profound. They are fundamentally concerned with the role of government in private affairs; accordingly, health taxes are tied to entrenched social values and morals.^1^ In this way, arguments over health taxes involve perceptions about caring for others, promoting fairness, contributing to society, preserving dignity and protecting freedoms. In this scoping review, we investigate factors that promote pro-tax and anti-tax policies by considering how social values and moral principles shape discussion of controversial issues and the related policy process for health taxes.

As countries continue to experiment with health taxes, it is timely to understand what drives national policy around health taxes. First, they are widely seen as an essential strategy for addressing the growing burden of non-communicable diseases (NCDs).^2^ Second, they can also generate sizeable revenues that can be dedicated to specific priorities for cash-strapped governments,^3^ including the UN’s Sustainable Development Goals.^4^ Third, markets are widely understood to be inefficient and many argue that the full cost of a commodity should be reflected in its price.^5^ Fourth, it is widely acknowledged that multinational corporations and the commercial determinants of health are responsible for accelerating NCDs, exacerbating socioeconomic inequalities, distorting regulatory environments and undermining public discourse.^6^ Fifth, many countries have a legacy of well-established health taxes and can point to their successes and failures.^3^ For these reasons, expansion of health taxes continues to be an alluring, although deeply political, prospect.

The recognition that health taxes extend to commodities beyond tobacco and alcohol has significantly amplified contestation in part because they involve products that are rarely conceived of as ‘bad’ or ‘sinful’.^7^ Sugar-sweetened beverage (SSBs) taxes, framed as a lucrative and easy way to eliminate excess calories, appears to be the leading edge of this movement. At least 54 countries have passed national or subnational SSB taxes.^8^ Health taxes that focus on nutrients such as sugar, salt and saturated fats as well as products such as ultra-processed foods affect retailers, distributors, suppliers, farmers and consumers. These industries are not only large and financially powerful but are also linked to other industries, including tobacco and alcohol.^9^ Much remains unknown about how large-scale changes to the nutrient profile of these products affect those responsible for producing them.^10^ Moreover, the food and beverage industry is seen as somewhat unique due to its privileged political position as producers of essential commodities, despite the need for strong regulatory oversight.^9 11^ As with the proliferation of SSBs, there is likely to be an increase in different types of nutrient and product taxes.^7^ While many of these new taxes have been concentrated in high-income countries, the emerging literature suggests health taxes are becoming more prevalent in low/middle-income countries (LMICs).^12^ For this to occur, however, more research is needed on the politics of this process.

Recent scholarship has shed light on the political dimensions of health tax policy. Robust evidence can be used, for example, to refute much of industry’s arguments about health taxes’ negative consequences.^3^ This has led to urgent calls for more research on the political economy of health taxes.^7^ Important themes related to the content, context and actors in health tax debates have been identified, underscoring the importance of process-related phenomena, such as framing.^12^ In this respect, media has an important role to play by mobilising frames around opposing values such as ‘market justice’ and ‘social justice’.^13^ Similarly, research in LMICs has identified three core frames of ‘pro-health’, ‘pro-economic’ and ‘fiscal scepticism’.^12^ Some argue that effective health tax policy is heavily tied to the success of resilient framing strategies deployed by advocates that, among other things, link revenue streams to policy proposals.^14 15^ Throughout this scholarship; however, researchers call for more research into the mechanics of this framing process.

Framing is an important, complicated and contested phenomenon in policy making.^16 17^ By selecting or omitting salient features of the social world, framing is an interactive way to generate shared understandings of both policy problems and solutions.^18 19^ In so doing, frames elicit strong emotions via the recruitment of deeply held morals and social values into the logics of political action.^20 21^ In this way, social psychologists have proposed that framing is a central means of using moral judgements to shape intuitions, resulting in social persuasion.^22^ Yet, the moral foundations of these judgements often remain obscure in much of the health policy framing literature.^23^ This includes the extent to which frames emphasise universal concerns such as fairness/cheating or loyalty/betrayal. Related to this, the goals to which individuals collectively strive, their social values, often remain tacit, when characterising policy processes.^24^ We propose that more research is needed to deconstruct framing dynamics to better understand the moral and social bases by which they resonate in situated health debates.

A focus on frames and the act of framing is particularly useful for understanding how health taxes are advanced and thwarted in specific contexts. Framing research on the policy process assumes many forms and has been conducted on several different health policy issues.^23^ Nevertheless, only recently have scholars turned their attention to framing as means of confronting corporate power.^25 26^ This is somewhat surprising given that industry thinks carefully about constructing frames to promote tobacco, alcohol or foods that are oily, salty and sweet, particularly through in-house marketing, public relations and executive teams as well as through contracted lobbyists and communications firms.^27^ Insight into industry actors’ ability to frame debates in ways that resonate with different values and morals reveals much about the nature of corporate political activity.^13 28^ Although some core frames have been identified in the health tax literature, the full range of arguments has yet to be sufficiently characterised. Similarly, many of the diverse rhetorical devices^29^ frames incorporate to move from cognitive reasoning to social persuasion remain poorly understood. A better understanding of how arguments are portrayed in framing contests is needed to understand how they define problems, diagnose causes, pass moral judgement and prescribe solutions.^30^ Moreover, the very act of framing changes how people view the issues under debate, the parties privy to the controversy and the process by which conflicts can be resolved.^31^ In this way, identifying the plurality and moral basis of pro-tax and anti-tax frames can help better illuminate how they resonate with different audiences. This is an important and often overlooked step in providing regulators, public health agencies, civil society and other advocates with effective strategies for limiting the industry’s authority and building support for better health tax proposals.

This article assesses the scope of framing scholarship on health taxes. This is important because these moral principles shape feelings about issues, which in turn guide thinking and action on them.^22^ This review highlights the unique ability of health taxes to simultaneously inspire and frustrate. By leveraging emotion in the policy process, they possess a distinct convening power and hold the potential to advance movements surrounding universal health coverage. Moreover, this review represents an attempt to understand more about the ways in which ideas influence the policy process and to provide recommendations for pro-tax health advocates. As such, quality appraisal was not pursued; instead, this review sought to identify common themes, highlight knowledge gaps and inform subsequent empirical research on framing health taxes.

## Methods

We conducted a scoping review using Arksey and O’Malley’s methodology for scoping reviews^32^ to understand how health taxes have been framed in policy debates (new unregistered protocol). This method includes: (1) identifying the research question, (2) identifying relevant studies, (3) study selection, (4) charting the data, (5) collating, summarising and reporting the results. The Preferred Reporting Items for Systematic Reviews and Meta-Analyses extension for Scoping Reviews guidelines were used (see online supplemental file 2). While a realist review may seem appropriate given our emphasis on theory and abductive reasoning, we concluded that the scoping review was better suited for reviewing the intricacies of implementing health interventions. We also considered a narrative review method but found it too unstructured to generate consistent insight. Thus, the scoping review method was determined to be flexible enough to accommodate a range of phenomena and structured enough to provide clear, balanced and replicable findings. See box 1 for search strategy and selection criteria.

10.1136/bmjgh-2023-012055.supp2 Supplementary data

Box 1 **Search strategy and selection criteria**

- Databases: SCOPUS, Web of Science, EMBASE, Pubmed, Proquest, Psychinfo.
- Sources: Journals, books, theses/dissertations.
- Search terms: see online supplemental file 1 for full search strategy. Terms were organised into three domains linked by Boolean “AND”: (1) product (eg, tobacco, alcohol, SSBs and so on), (2) instrument (ie, tax), framing (ie, Fram* NOT framework).
- Dates: January 2000 to November 2022.
- Language: English.
- Inclusion criteria: (1) directly concerns unhealthy commodities, (2) ‘Framing’, ‘frames’ or similar mentioned in abstract, (3) article concerns the policy process or media constructions, (4) original (empirical) research and (5) English language abstract available.
- Exclusion criteria: (1) published before 2000, (2) reviews, editorials, conference abstracts or commentaries, (3) modelling studies, (4) insufficient analysis of framing and (5) does not concern a health tax.

10.1136/bmjgh-2023-012055.supp1 Supplementary data

Six databases were searched in August 2021 and November 2022 using terms categorised into five domains: product (eg, tobacco, alcohol, SSBs and so on), instrument (ie, tax), policy actor (eg, industry, government, civil society, non-governmental organization (NGO) and so on), process (eg, argument, debate, lobbying and so on) and framing (ie, Fram* NOT framework). After experimentation, two further modifications were made. First, the five categories were collapsed into three (product, instrument, framing), which made the search results more manageable and more accurate (eg, by including pre-identified ‘tracer’ articles). Second, we expanded the list of terms in the product category to include those used in a similar review.^12^ Please see Appendix A in online supplemental file 1 for the full search strategy. No language or date restrictions were applied (neither by database search entries nor by filters), and results were limited to studies with abstracts. Database searches were performed in English. Covidence was used to organise, screen and review all articles.

This review adopted the same screening and review strategy used in previous research.^33^ Titles and abstracts were screened by ADK and a research assistant to identify those with each of the three domains present in the title or abstract. All full-text articles were also screened independently by both reviewers. Covidence flags conflicts, when reviewers make different decisions about whether to accept/reject an article or differ in their reasons for exclusion. Reviewers discussed these conflicts before reaching a joint determination when necessary. Included abstracts were often vague and the links between framing and the health tax were not required to be obvious. Articles were excluded if they were published before 2000; were review articles, editorials, conference abstracts or commentaries; were modelling studies; did not involve framing; or did not concern a health tax.

Data charting was pursued by ADK and a research assistant by completing a Google form for each article. At the time, this was considered optimal because Covidence’s data extraction tool did not allow reviewers to select multiple items within a category (they have since changed this). Descriptive information about each article was captured by each researcher, discussed, and mutual agreement reached for information such as the study design, actors, taxes, frames, arguments, outcomes and corporate political activity.^27^ In addition to this, Haidt’s moral foundations^34^ (six dualities including care/harm, fairness/cheating, liberty/oppression, sanctity/degradation, authority/subversion and loyalty/betrayal) and Stone’s social values^24^ (five constructs including equity, efficiency, welfare, liberty and security) were interpreted in pro-tax and anti-tax framing strategies, in the same deliberative manner. Charted data from the Google form were exported to Microsoft Excel, where it was transformed into pivot tables by ADK to identify trends. Patients and/or the public were not involved in the design, conduct or reporting of this research. We plan to engage the public in dissemination as part of the Alliance for Health Policy and Systems Research/WHO’s broader health tax portfolio of work.

## Results

**Figure 1:**
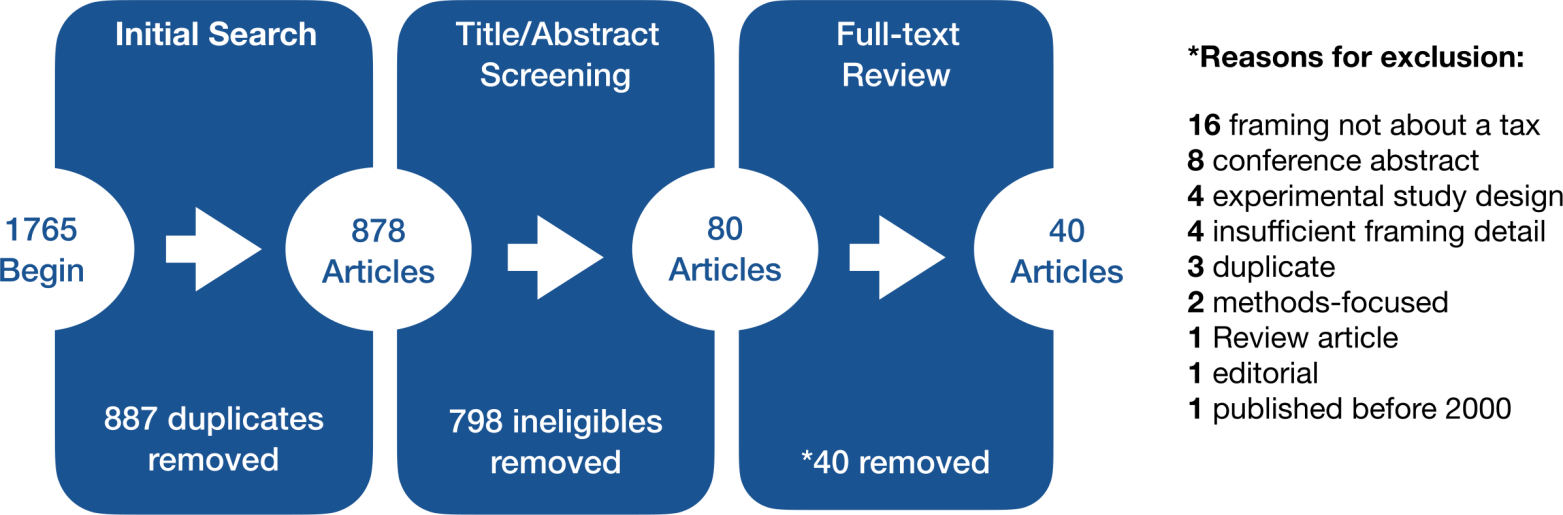
Flow diagram.

A total of 1765 articles were returned from the initial 2021 search and an updated search in 2022, with 878 remaining after removing duplicates. Title and abstract screening further narrowed the number of articles to 80. Following a full-text review, 40 articles were removed, most commonly because framing did not concern a tax specifically. A total of 40 articles were included in the final analysis (see figure 1 and Appendix B). Please see online supplemental file 3 for summary tables.

10.1136/bmjgh-2023-012055.supp3 Supplementary data

Research on framing health taxes is growing rapidly but remains relatively scarce, concentrated on high-income countries and limited in scope. Articles ranged from 2006 to 2021, with over half (56%) published in the last 3 years (2022, n=4; 2021, n=13; 2020, n=5). Almost all studies focused on a single country, with just two^35 36^ comparing across countries. Many articles (n=17) reported more than one data source. There was no dominant theoretical lineage for the pool of articles. One dissertation^37^ was included. The rest were peer-reviewed articles spread relatively evenly across 32 different journals.

The administrative level at which health taxes are levied is increasingly varied, particularly in the USA (see table 1). While most studies focused on national taxes (n=27), increasingly taxes on SSBs focused were sub-national. This experience suggests advocates are increasingly developing grassroots campaigns to avoid the concentrated corporate influence on national-level policymakers. At the same time, the industry proactively shifts venues to higher jurisdictions to pre-empt the adoption of health taxes at lower jurisdictions.^38^ For example, the USA state of California recently passed a law banning further SSBs taxes after four municipalities within the state enacted them.^39^ These measures are often voted on by smaller representative bodies, such as city councils, or via ballot items. Framing occurs both in the lead-up to a vote as well as the industry’s efforts to word ballot items in specific ways.^40^ This perhaps explains the preponderance of specific slogans and catchphrases in media campaigns.

**Table 1:** Descriptive overview, by category with >1 study

	Total (n, %)
Country	
USA	17 (43)
Australia	4 (10)
UK	2 (5)
Mexico	2 (5)
South Africa	2 (5)
Administrative level	
National	27 (68)
City	9 (23)
State	8 (20)
County	3 (8)
Data source	
News media	28 (70)
Interviews	14 (35)
Government documents	10 (25)
Industry documents	7 (18)
NGO reports	5 (13)
Legislative proceedings	3 (8)
Social media	2 (5)
Commodity (taxed)	
SSBs	23 (58)
Tobacco	12 (30)
Alcohol	7 (18)
Tax type	
Excise	30 (75)
VAT	7 (18)
Retail transaction	2 (5)
Outcome	
Old tax modified	14 (35)
New tax created	14 (35)
Contestation (unresolved)	12 (30)
New tax rejected	5 (13)
New tax modified	2 (5)

*Individual categories may be represented more than once in some studies (eg, multicountry, multidata sources, multitax and so on).

NGO, Non-governmental Organization; SSBs, sugar-sweetened beverages; VAT, Value-Added Tax.

Framing research on health taxes has been particularly influenced by the recent growth of SSBs. This commodity was the most popular (n*=*23), followed by tobacco (n=12) and alcohol (n=7) taxes. Few studies (n=5) compared multiple commodities subject to taxation in a single context. The majority (75%) were reported to be excise taxes (n=30), though this was occasionally difficult to identify. Similarly, the name of the health tax varied considerably as did the level of taxation, which was usually volumetric. The outcome of the framing contests varied, with the most common being an old tax that was modified (n=14), or a new tax created (n=14), unresolved contestation (n=12), a new tax rejected (n=5), or a new tax modified (n*=*2). It is important to note that these outcomes were at the time of writing, as reported by the authors.

Actors engaged in framing were diverse and engaged in other types of corporate political activity (see table 2). A range of categories of actors (low n=1, high n=12) were represented in individual articles. Civil society, including NGOs, industry associations/front groups and the media were present in most articles. While government, in the form of ministries/departments of health, legislators and (to a lesser extent) ministries of finance/treasury were also commonly represented. Professional associations were scarcely represented and largely peripheral. Jingles and slogans were particularly prevalent in media campaigns for/against health taxes as subnational ballot measures.

**Table 2:** Actors and frames

	Total (n, %)
Organisation (>10)	
NGOs	12 (80)
Industry association/front group	30 (75)
Corporation	27 (68)
Ministry/Department of Health	26 (65)
Legislative branch	21 (53)
Media	20 (49)
Academic institution	16 (40)
Ministry of Finance/treasury	15 (38)
Executive branch	15 (38)
Corporate political activity	
Shape evidence via lobbying	29 (73)
Shape evidence via research funding priorities	8 (20)
Constituency-building via non-core activities	8 (20)
Constituency-building via partnership with charities	7 (18)
Policy process via consultation or law drafting	6 (15)
Policy process via voluntary agreements	5 (13)
Frame type	
Policy action (surface level)	30 (75)
Institutional (intermediate level)	10 (25)
Metacultural (deep/broad level)	4 (10)
Frames (top 3)	
Health	19 (48)
Revenue generation	15 (38)
Economy	13 (33)

NGO, Non-Governmental Organization.

There was a considerable degree of variation in how health tax frames were identified and presented in the literature. Multiple frames were present in analyses of health taxes. Articles featured a range of frames (low n=1, high n=21) within them. Following Rein and Schön’s characterisation,^41^ we located frames at different levels of abstraction, with the majority, by nature of our focus on health taxes, dedicated to surface-level policy action frames (n=30). Because framing is an intersubjective situated phenomenon, researchers characterise them by either interpreting their broader elements or presenting them as data unsynthesised. To facilitate comparison, we identified the most common three groupings across the 35 articles: health (n=19), revenue generation (n=15) and broader economic (n=13) frames. While health frames (used by pro-tax advocates) and economic frames (used by anti-tax opponents) were prevalent throughout, the relative priority of each and their causal influence on outcomes was often a source of debate among researchers.

The moral basis for pro-tax and anti-tax framing differs in potentially important ways. First, it should be noted that most articles did not explicitly link frames to moral foundations or social values (see table 3). We interpreted them, however, which yielded differences between the two groups. Health tax advocates most frequently drew on moral principles of care/harm (n=35) and loyalty/betrayal (n=5) was the least likely. Similarly, advocates were understood to base their arguments on social values such as welfare (n=34), and least commonly, security (n=11). Opponents of health taxes appeared to use a more diverse, and less pronounced, set of moral foundations, with fairness/cheating (n=26) the most common and sanctity/degradation (n=4) the least common. Opponents similarly touched on social values more consistently, with liberty (n=21) and efficiency (n=21) the most common and, like the advocates, security (n=12) the least common. A single study^37^ covered nearly all moral foundations and social values. Interpreting and disentangling moral principles and social values was difficult (see below); however, the literature does seem to suggest that anti-tax coalitions strategically deploy a morally more diverse array of frames in policy contests. While pro-tax frames disproportionately focused on principles of care/harm, anti-tax frames appear to be slightly more evenly distributed, with fairness/cheating being the leading concern. This trend holds when moving from moral principles to the social values that underpin them.

**Table 3:** Morals and values, by tax position (# articles, % total)

	Pro-tax	Anti-tax
Moral foundations		
Care/harm	35 (88)	18 (45)
Fairness/cheating	18 (45)	26 (65)
Liberty/oppression	17 (43)	21 (53)
Sanctity/degradation	10 (25)	4 (10)
Authority/subversion	7 (18)	10 (25)
Loyalty/betrayal	5 (13)	8 (20)
Social values		
Welfare	34 (85)	21 (53)
Equity	21 (53)	21 (53)
Efficiency	21 (53)	19 (48)
Liberty	16 (40)	19 (48)
Security	11 (28)	12 (30)

Analysis of the number and types of arguments presented in these articles was fruitful (see table 4). We mapped readily identifiable arguments in anticipation of the aforementioned challenges with analysing frames in this literature. We developed a list of eight common arguments used for and against taxation. Building iteratively from a pre-set list was instructive given that most frames were surface-level policy action frames that present themselves as arguments. In fact, researchers often used the two concepts (frames and arguments) interchangeably. Pro-tax arguments focused on the ability of health taxes to reduce morbidity and mortality (n=33), generate government revenue (n=22), save healthcare costs (n=19), among other things. Anti-tax arguments characterised health taxes as a threat to industry (n=25), tax on the poor (n=23), harming jobs (n=21) and more. Similar to the moral foundations and social values, anti-tax arguments were possibly more diversified and less concentrated on a few themes.

**Table 4:** Arguments for health taxes (# articles, % total)

Pro-tax argument	Total (n, %)	Total (n, %)	Anti-tax argument
Reduce suffering, death	33 (83)	25 (63)	Threat to industry
Lucrative for governments	22 (55)	23 (58)	Tax on the poor
Cost containment/savings	19 (48)	21 (53)	Hurts/eliminates jobs
Pro-poor policy	14 (35)	21 (53)	Better means to end
Education funding	9 (23)	19 (48)	Narrow and unfair
Everyone else is doing it	9 (23)	18 (45)	Meaningless (too small/ineffective)
Product reformation	5 (13)	17 (43)	Nanny state
Cheap	4 (10)	5 (13)	Promotes illicit trade

## Discussion

The literature on framing health taxes points to several different trends. Research on framing health taxes is increasing rapidly, especially in high-income countries, and particularly on SSBs. Less is known about how health taxes, particularly for foods other than SSBs, are framed in LMICs. Moreover, little cross-country research has been conducted. Most research focuses on the adoption of a new tax that incorporates a variety of different consideration based on the interplay of interests from multiple actors. More evidence is needed to explore how framing modifies existing health taxes, particularly at sub-national levels.

There is some evidence to suggest that grassroots health tax advocates may be better positioned to develop arguments that resonate with local constituents.^40 42^ This includes portraying the industry as nefarious outsiders meddling in local affairs. Despite this, however, moral principles such as loyalty/betrayal or even authority/subversion associated with ‘local pride’ are rarely leveraged in frames by health advocates. More research is needed to explore whether bringing these principles to the fore helps pro-tax frames resonate with key constituents.

Our finding that anti-tax coalitions may adopt framing strategies that potentially incorporate a more diverse set of morals and values was surprising. This is perhaps attributable to the fact that corporations are uniquely skilled at framing; to sell consumer products, corporations develop sophisticated marketing, branding and advertising strategies, and invest sizeable resources in public relations firms to shape regulatory environments. Despite this, there are inherent gaps in the moral foundations of their framing strategies. One conspicuous gap that also appears to be underused by pro-tax frames is related to sanctity/degradation. Some evidence from successful SSBs tax campaigns in Mexico,^43^ where graphic images of amputations were displayed on billboards, and in Philadelphia,^44^ where doctors delivered public testimonials about diabetes patients with amputations, demonstrate this potential. Moreover, the experience of graphic warning labels on cigarette packages and in road safety campaigns further underscores the persuasive effect of appeals to sanctity/degradation, and the difficulty industry faces in challenging them. More research is needed to determine whether a greater diversity of frames and emphasis on specific moral dimensions can influence policy outcomes across contexts.

Media campaigns, essentially a strategic framing exercise, are expensive, but often short. The literature is clear that corporations, often through trade associations and front groups, invest heavily in media campaigns to undermine health tax proposals.^13^ A number of articles, however, noted the success of private philanthropic organisations, particularly Bloomberg Philanthropies, in advancing health taxes in Berkeley, CA^42^; Philadelphia, PA^45^; Mexico^35 43^ and India.^46^ In fact, tactics supported by Bloomberg Philanthropies resemble those deployed to confront health-harming industries during Michael Bloomberg’s tenure as Mayor of New York City.^47^ Much of the costs for advocates and opponents are used for evidence generation, public relations firms and consultants to develop frames that resonate with constituents. While these campaigns are intense, they are relatively short, and some evidence suggests that health advocates benefit from prolonged media exposure which normalises the tax discourse.^40^ More research is needed to determine whether the length and intensity of debate about health taxes affect policy outcomes across contexts.

The formal absence of health professional associations (mentioned peripherally in just seven articles) in these debates is somewhat surprising. Moreover, virtually none of the major health national professional associations were named, with the limited presence accounted for by smaller specialty societies or related advocacy groups. Individual doctors and nurses were mentioned in fewer articles and in support of^36^ and opposition to^48^ regulation. This is somewhat surprising as ministry/department of health officials were central actors in framing health taxes. One explanation for the absence of health professional associations, and the medical profession in particular, is that this simply reflects broader trends of declining political legitimacy for the medical profession in the USA^49^ (which accounted for 43% of articles), though the opposite may be true elsewhere.^50^ More research is needed to understand the limited role of health professional associations in framing health taxes and whether this can/should change

Findings were mixed about how pro-tax advocates should present the revenue generation potential of health taxes in policy debates. Despite the problematic nature of earmarking and public administration concerns about unstable revenue streams, how revenue generated from taxes will be used appears to be a central consideration for voters and other constituents.^51^ In some contexts, researchers argue that details about how revenue will be generated leaves pro-tax advocates open to a number of arguments by anti-tax opponents, often drawing on legacies of well-intentioned, but misguided social welfare programmes that widen inequalities, waste resources or have negligible effects.^40^ For others, strategically emphasising dedicated revenue streams for programmes that have wider appeal, such as funding early education initiatives, is crucial for health tax frame resonance.^44 45^ What seems clear from this review; however, is that careful framing of revenue seems to have an important bearing on health tax debates. Framing that focuses disproportionately on the health benefits of taxes, for example, is unlikely to be sufficient in many policy settings. More research is needed to fully understand how best to position the revenue implications of health tax frames, including their levels of specificity and targeting mechanisms.

Methodologically, it may be useful to start future framing research with an inventory of pro-tax and anti-tax arguments from previous media analyses. One article^52^ provided a useful taxonomy of over 40 pro-tax and anti-tax arguments grouped into 10 and 11 themes. Starting with them provides the analyst something to grab onto when trying to make sense of confusing and abstract phenomena. Disaggregating these frames/arguments into their ‘signature elements’^53^ as a subsequent step is important for looking at their persuasive power. After doing this, researchers are in a better position to think about how frames coalesce into broader meta-cultural narratives. In this way, we hope to provide practical guidance for scholars interested in pursuing work in this area.

Articles represent a variety of approaches for analysing framing; they are epistemologically eclectic. For example, some approaches can involve computational measures,^52^ historical narratives^54^ or rhetorical deconstruction.^55^ These differences are deeper than deployment of methods or the use of well-established theoretical heuristics and reflect the power of ideas. For this reason, researchers looking for a uniquely persuasive framing, that is, universally applicable are likely to be disappointed. No single frame is better or worse, but rather certain frames resonate in specific contexts. Analysis can help characterise the messy plurality of frames and their interactions. One noteworthy piece of work in this review used news media, advocacy and tobacco industry documents to compare virtually all types of arguments, clearly touching on each of the moral foundations, social values of interest and frame interaction to explain differential policy outcomes.^37^ More, in-depth research using a variety of different data sources is needed in other contexts.

This study had multiple limitations. First, we were unable to include many fossil fuels taxes in our review. While one article on a gas tax was included, we suspect that more framing work on fossil fuels taxes might exist; however, we were unable to capture it, perhaps because much of the focus has been on carbon taxes which do not focus on consumption. Second, we excluded research before 2000, when many tobacco and alcohol taxes were passed, which perhaps explains the preponderance of newer research on commodities such as SSBs. Third, we found it surprisingly difficult to consistently capture the frames authors presented in some circumstances because they were focused at different levels of abstraction. Pre-selecting frames as we did with arguments might have made this task easier than collecting what authors reported their frames to be. Fourth, we found our list of 16 tax arguments to be insufficient. While others^52^ provide a more extensive taxonomy, we noted that even this did not cover broader political strategies. Fifth, we found it difficult to consistently code moral principles and social values. For this reason, both AK and a research assistant were responsible for coding all morals and values and discussing differences of opinion. Sixth, the distinction between arguments and frames and morals and values were not always clear and often difficult to disentangle. Future research would be better to focus on one of each pair instead of attempting to capture or interpret all simultaneously. Seventh, we excluded a significant number of experimental studies that ‘test’ frames in early rounds of screening. While this information is undoubtedly useful, we found it difficult to accommodate this insight given that we were focused on how different frames conflict and compete in political settings. Finally, we only included studies with ‘fram*’ in the abstract. Many articles that concerned broader regulation of relevant commodities may have analysed frames, but because they did not mention this specifically in the abstract, were not included.

The strengths of this study were multiple. By bringing together the health policy literature on health taxes and framing, we have provided insight into a topic of immediate relevance to research and public health practice. Moreover, this work can inform subsequent empirical research to fill these gaps, particularly in LMICs. We anticipate that cross-country work, informed by this review, can also help contribute to a pragmatic framework for studying and applying frames in health policy arenas.

## Conclusions

The rapid growth of research on framing health taxes reflects a global trend of increased efforts, particularly in the wake of the costly COVID-19 pandemic, to regulate the consumption of harmful consumer products and raise government revenues. This process involves divergent actors who actively engage in framing as means of social persuasion. Scope remains for incorporating a broader array of morals and values into frames, especially for health advocates. Nevertheless, no single frame appears to be uniquely effective across all settings, rather the situated interaction of multiple frames over time influences the policy process. More in-country comparative research, using different types of data, is needed to understand the mechanics of frames and the social processes by which they can be persuasive. Research on framing health taxes can advance theory about collective sensemaking processes. More importantly, however, these insights can help constrain disproportionate corporate political influence, rebalance global patterns of unfair consumption and ultimately enhance the health and happiness of future generations.

## Data Availability

All data relevant to the study are included in the article or uploaded as supplementary information.
