# Supplementary material for: Framing health taxes: a scoping review
Source: BMJ Glob Health. 2023 Oct 9;8(Suppl 8):e012055. doi: 10.1136/bmjgh-2023-012055 (PMC10565303; doi:10.1136/bmjgh-2023-012055)
Supplement: Supplementary data [file bmjgh-2023-012055supp001.pdf]

Supplemental Material File 1

Appendix A. Search Strategy

SCOPUS : 429

(TITLE-ABS-KEY ((tobacco OR smoke OR cigarette OR cigar OR alcohol OR "alco-pop" OR beer OR wine OR whiskey OR spirits OR "Sugar-sweetened beverage" OR "Sweetened beverage" OR "carbonated beverage" OR "SSB" OR "Soda" OR "soft drink" OR "softdrink" OR "Sin" OR "Health" OR "Excise" OR "Consumption" OR "junk food" OR "high salt" OR "high sodium" OR "calorie-dense" OR "salt reduc*" OR "Sodium reduc*" OR "high fat" OR "saturated fat" OR "energy dense" OR "unhealthy commodit*" OR "harmful corporat*" OR "ultra processed food" OR "trans-fat" OR "trans fat" OR "diet*" OR "lifestyle-related" OR "commercial determinant*" OR "food industry*" OR "fossil" OR "fuel" OR "Gas" OR "natural gas" OR "oil" OR "coal" OR "carbon"))) AND (TITLE-ABS-KEY (("Tax" OR "Hypothecat*" OR "Earmark*" OR "Fiscal"))) AND (TITLE-ABS-KEY ((fram* AND NOT "Framework")))

WEB OF SCIENCE: 377

TS= ((Tobacco OR smoke OR Cigarette OR Cigar OR Alcohol OR "alco-pop" OR Beer Or Wine Or Whiskey Or Spirits OR "Sugar-sweetened beverage" OR "Sweetened beverage" OR "carbonated beverage" OR "SSB" OR "Soda" OR "soft drink" OR "softdrink" OR "Sin" OR "Health" OR "Excise" OR "Consumption" OR "junk food" OR "high salt" OR "high sodium" OR "calorie-dense" OR "salt reduc*" OR "sodium reduc*" OR "high fat" OR "saturated fat" OR "energy dense" OR "unhealthy commodit*" OR "harmful corporat*" OR "ultra processed food" OR "trans-fat" OR "trans fat" OR "diet*" OR "lifestylerelated"OR "commercial determinant*" OR "food industry*" OR "fossil" OR "fuel" OR "Gas" OR "natural gas" OR "oil" OR "coal" OR "carbon"))
(("Tax" OR "Hypothecat*" OR "Earmark*" OR "Fiscal"))
(Fram* NOT "Framework")

EMBASE: 266

'tobacco':ti,ab,kw OR smoke:ti,ab,kw OR cigarette:ti,ab,kw OR cigar:ti,ab,kw OR alcohol:ti,ab,kw OR 'alco pop':ti,ab,kw OR beer:ti,ab,kw OR wine:ti,ab,kw OR whiskey:ti,ab,kw OR spirits:ti,ab,kw OR 'sugar-sweetened beverage':ti,ab,kw OR 'sweetened beverage':ti,ab,kw OR 'carbonated beverage':ti,ab,kw OR ssb:ti,ab,kw OR soda:ti,ab,kw OR 'soft drink':ti,ab,kw OR softdrink:ti,ab,kw OR sin:ti,ab,kw OR health:ti,ab,kw OR excise:ti,ab,kw OR consumption:ti,ab,kw OR 'junk food':ti,ab,kw OR 'high salt':ti,ab,kw OR 'high sodium':ti,ab,kw OR 'calorie dense':ti,ab,kw OR 'salt reduc*':ti,ab,kw OR 'sodium reduc*':ti,ab,kw OR 'high fat':ti,ab,kw OR 'saturated fat':ti,ab,kw OR 'energy dense':ti,ab,kw OR 'unhealthy commodit*':ti,ab,kw OR 'harmful corporat*':ti,ab,kw OR 'ultra processed food':ti,ab,kw OR 'trans fat':ti,ab,kw OR 'trans fat':ti,ab,kw OR diet*:ti,ab,kw OR 'lifestyle related':ti,ab,kw OR 'commercial determinant*':ti,ab,kw OR 'food industry*':ti,ab,kw OR ‘fossil’:ti,ab,kw OR ‘fuel’:ti,ab,kw OR ‘Gas’:ti,ab,kw OR ‘natural gas’:ti,ab,kw OR ‘oil’:ti,ab,kw OR ‘coal’:ti,ab,kw OR ‘carbon’:ti,ab,kw

Koon AD, Marten R. *BMJ Glob Health* 2023; 8:e012055. doi: 10.1136/bmjgh-2023-012055

tax:ti,ab,kw OR 'hypothecat*':ti,ab,kw OR 'earmark*':ti,ab,kw OR fiscal:ti,ab,kw
fram*:ti,ab,kw NOT 'framework':ti,ab,kw

PUBMED: 213

"tobacco"[MeSH Terms] OR "tobacco"[All Fields] OR "tobacco products"[MeSH Terms] OR ("tobacco"[All Fields] AND "products"[All Fields]) OR "tobacco products"[All Fields] OR "tobaccos"[All Fields] OR "tobacco s"[All Fields] OR ("smoke"[MeSH Terms] OR "smoke"[All Fields] OR "smoke s"[All Fields] OR "smoked"[All Fields] OR "smokes"[All Fields] OR "smoking"[MeSH Terms] OR "smoking"[All Fields] OR "smokings"[All Fields] OR "smoking s"[All Fields]) OR ("cigarett"[All Fields] OR "cigarette s"[All Fields] OR "cigaretts"[All Fields] OR "tobacco products"[MeSH Terms] OR ("tobacco"[All Fields] AND "products"[All Fields]) OR "tobacco products"[All Fields] OR "cigarette"[All Fields] OR "cigarettes"[All Fields]) OR ("tobacco products"[MeSH Terms] OR ("tobacco"[All Fields] AND "products"[All Fields]) OR "tobacco products"[All Fields] OR "cigar"[All Fields] OR "cigars"[All Fields]) OR ("alcohol s"[All Fields] OR "alcoholate"[All Fields] OR "alcoholates"[All Fields] OR "alcoholic s"[All Fields] OR "alcoholics"[MeSH Terms] OR "alcoholics"[All Fields] OR "alcoholic"[All Fields] OR "alcoholism"[MeSH Terms] OR "alcoholism"[All Fields] OR "alcoholisms"[All Fields] OR "alcoholism s"[All Fields] OR "alcoholization"[All Fields] OR "alcohols"[MeSH Terms] OR "alcohols"[All Fields] OR "ethanol"[MeSH Terms] OR "ethanol"[All Fields] OR "alcohol"[All Fields]) OR (("beer"[MeSH Terms] OR "beer"[All Fields]) AND "Or"[All Fields] AND ("wine"[MeSH Terms] OR "wine"[All Fields]) AND "Or"[All Fields] AND ("whiskey"[All Fields] OR "whiskeys"[All Fields]) AND "Or"[All Fields] AND ("spirit"[All Fields] OR "spirited"[All Fields] OR "spiritism"[All Fields] OR "spirits"[All Fields])) OR "Sugar-sweetened beverage"[All Fields] OR "Sweetened beverage"[All Fields] OR "carbonated beverage"[All Fields] OR "SSB"[All Fields] OR "Soda"[All Fields] OR "soft drink"[All Fields] OR "softdrink"[All Fields] OR "Sin"[All Fields] OR "Health"[All Fields] OR "Excise"[All Fields] OR "Consumption"[All Fields] OR "junk food"[All Fields] OR "high salt"[All Fields] OR "high sodium"[All Fields] OR "calorie-dense"[All Fields] OR "salt reduc*"[All Fields] OR "sodium reduc*"[All Fields] OR "high fat"[All Fields] OR "saturated fat"[All Fields] OR "energy dense"[All Fields] OR "unhealthy commodit*"[All Fields] OR (("harmed"[All Fields] OR "harmful"[All Fields] OR "harmfulness"[All Fields] OR "harming"[All Fields] OR "harms"[All Fields]) AND "corporat*"[All Fields]) OR "ultra processed food"[All Fields] OR "trans-fat"[All Fields] OR "trans-fat"[All Fields] OR "diet*"[All Fields] OR "lifestyle-related"[All Fields] OR "commercial determinant*"[All Fields] OR "food industry*"[All Fields] OR "fossil"[All Fields] OR "fuel"[All Fields] OR "Gas"[All Fields] OR "natural gas"[All Fields] OR "oil"[All Fields] OR "coal"[All Fields] OR "carbon"[All Fields]

Translations
Tobacco: "tobacco"[MeSH Terms] OR "tobacco"[All Fields] OR "tobacco products"[MeSH Terms] OR ("tobacco"[All Fields] AND "products"[All Fields]) OR "tobacco products"[All Fields] OR "tobaccos"[All Fields] OR "tobacco's"[All Fields]
smoke: "smoke"[MeSH Terms] OR "smoke"[All Fields] OR "smoke's"[All Fields] OR "smoked"[All Fields] OR "smokes"[All Fields] OR "smoking"[MeSH Terms] OR "smoking"[All Fields] OR "smokings"[All Fields] OR "smoking's"[All Fields]
Cigarette: "cigarett"[All Fields] OR "cigarette's"[All Fields] OR "cigaretts"[All Fields] OR "tobacco products"[MeSH Terms] OR ("tobacco"[All Fields] AND "products"[All Fields]) OR "tobacco products"[All Fields] OR "cigarette"[All Fields] OR "cigarettes"[All Fields]

Koon AD, Marten R. *BMJ Glob Health* 2023; 8:e012055. doi: 10.1136/bmjgh-2023-012055

Cigar: "tobacco products"[MeSH Terms] OR ("tobacco"[All Fields] AND "products"[All Fields]) OR "tobacco products"[All Fields] OR "cigar"[All Fields] OR "cigars"[All Fields]
Alcohol: "alcohol's"[All Fields] OR "alcoholate"[All Fields] OR "alcoholates"[All Fields] OR "alcoholic's"[All Fields] OR "alcoholics"[MeSH Terms] OR "alcoholics"[All Fields] OR "alcoholic"[All Fields] OR "alcoholism"[MeSH Terms] OR "alcoholism"[All Fields] OR "alcoholisms"[All Fields] OR "alcoholism's"[All Fields] OR "alcoholization"[All Fields] OR "alcohols"[MeSH Terms] OR "alcohols"[All Fields] OR "ethanol"[MeSH Terms] OR "ethanol"[All Fields] OR "alcohol"[All Fields]
Beer: "beer"[MeSH Terms] OR "beer"[All Fields]
Wine: "wine"[MeSH Terms] OR "wine"[All Fields]
Whiskey: "whiskey"[All Fields] OR "whiskeys"[All Fields]
Spirits: "spirit"[All Fields] OR "spirited"[All Fields] OR "spiritism"[All Fields] OR "spirits"[All Fields] OR "fossil"[All Fields] OR "fuel"[All Fields] OR "Gas"[All Fields] OR "natural gas"[All Fields] OR "oil"[All Fields] OR "coal"[All Fields] OR "carbon"[All Fields]

"Tax"[All Fields] OR "hypothecat*"[All Fields] OR "earmark*"[All Fields] OR "Fiscal"[All Fields]

"fram*"[All Fields] NOT "Framework"[All Fields]

PROQUEST: 365 (just articles; 140 of dissertations/thesis, books excluded)

noft(((Tobacco OR smoke OR Cigarette OR Cigar OR Alcohol OR "alco-pop" OR Beer Or Wine Or Whiskey Or Spirits OR "Sugar-sweetened beverage" OR "Sweetened beverage" OR "carbonated beverage" OR "SSB" OR "Soda" OR "soft drink" OR "softdrink" OR "Sin" OR "Health" OR "Excise" OR "Consumption" OR "junk food" OR "high salt" OR "high sodium" OR "calorie-dense" OR "salt reduc*" OR "sodium reduc*" OR "high fat" OR "saturated fat" OR "energy dense" OR "unhealthy commodit*" OR "harmful corporat*" OR "ultra processed food" OR "trans-fat" OR "trans fat" OR "diet*" OR "lifestyle-related" OR "commercial determinant*" OR "food industry*" OR “fossil" OR "fuel" OR "Gas" OR "natural gas" OR "oil" OR "coal" OR "carbon")))
noft((("Tax" OR "Hypothecat*" OR "Earmark*" OR "Fiscal")))
noft((Fram* NOT "Framework"))

PSYCINFO: 115 (includes 16 dissertations/thesis, 9 books)

(Tobacco OR smoke OR Cigarette OR Cigar OR Alcohol OR "alco-pop" OR Beer Or Wine Or Whiskey Or Spirits OR "Sugar-sweetened beverage" OR "Sweetened beverage" OR "carbonated beverage" OR "SSB" OR "Soda" OR "soft drink" OR "softdrink" OR "Sin" OR "Health" OR "Excise" OR "Consumption" OR "junk food" OR "high salt" OR "high sodium" OR "calorie-dense" OR "salt reduc*" OR "sodium reduc*" OR "high fat" OR "saturated fat" OR "energy dense" OR "unhealthy commodit*" OR "harmful corporat*" OR "ultra processed food" OR "trans-fat" OR "trans fat" OR "diet*" OR "lifestyle-related" OR "commercial determinant*" OR "food industry*" OR "fossil" OR "fuel" OR "Gas" OR "natural gas" OR "oil" OR "coal" OR "carbon")
("Tax" OR "Hypothecat*" OR "Earmark*" OR "Fiscal")
(Fram* NOT "Framework")

Appendix B. List of Articles Included in Review

Ahaibwe, Gemma, Safura Abdool Karim, Anne-Marie Thow, Agnes Erzse, and Karen Hofman. 2021. "Barriers to, and Facilitators of, the Adoption of a Sugar Sweetened Beverage Tax to Prevent Non-Communicable Diseases in Uganda: A Policy Landscape Analysis." *GLOBAL HEALTH ACTION* 14 (1). https://doi.org/10.1080/16549716.2021.1892307.

Akin-Onitolo, A., and B. Hawkins. 2021. "Framing Tobacco Control: The Case of the Nigerian Tobacco Tax Debates." *Health Policy and Planning* in press.

Anaf, J, M Fisher, E Handsley, F Baum, and S Friel. 2021. "'Sweet Talk': Framing the Merits of a Sugar Tax in Australia." *Health Promotion International*, no. (Anaf J.; Fisher M.; Baum F.) Southgate Institute for Health, Society and Equity, College of Medicine and Public Health, Flinders University, Sturt Rd ,Bedford Park, Australia(Handsley E.) School of Law, Western Sydney University, Locked Bag 1797, Penrith. https://doi.org/10.1093/heapro/daaa152.

Asada, Yuka, Sabira Taher, Andrea Pipito, and Jamie F Chriqui. 2021. "Media Coverage and Framing of Oakland's Sugar-Sweetened Beverage Tax, 2016-2019." *AMERICAN JOURNAL OF HEALTH PROMOTION* 35 (5): 698–702. https://doi.org/10.1177/0890117120986104.

Balbach, Edith D, Abby Herzberg, and Elizabeth M Barbeau. 2006. "Political Coalitions and Working, Women: How the Tobacco Industry Built a Relationship with the Coalition of Labor Union Women." *JOURNAL OF EPIDEMIOLOGY AND COMMUNITY HEALTH* 60 (2): 27–32. https://doi.org/10.1136/jech.2006.046276.

Bridge, G, S W Flint, and R Tench. 2020. "An Exploration of the Portrayal of the UK Soft Drinks Industry Levy in UK National Newspapers." *Public Health Nutrition* 23 (17 PG-3241–3249): 3241–49. https://doi.org/10.1017/S1368980020000208.

Buckton, Christina H, Chris Patterson, Lirije Hyseni, S Vittal Katikireddi, Ffion Lloyd-Williams, Alex Elliott-Green, Simon Capewell, and Shona Hilton. 2018. "The Palatability of Sugar-Sweetened Beverage Taxation: A Content Analysis of Newspaper Coverage of the UK Sugar Debate." *PLOS ONE* 13 (12). https://doi.org/10.1371/journal.pone.0207576.

Campbell, Norah, Melissa Mialon, Kathryn Reilly, Sarah Browne, and Francis M Finucane. 2020. "How Are Frames Generated? Insights from the Industry Lobby against the Sugar Tax in Ireland." *SOCIAL SCIENCE \& MEDICINE* 264 (November). https://doi.org/10.1016/j.socscimed.2020.113215.

Campbell, Richard B, and Edith D Balbach. 2009. "Building Alliances in Unlikely Places: Progressive Allies and the Tobacco Institute's Coalition Strategy on Cigarette Excise Taxes." *AMERICAN JOURNAL OF PUBLIC HEALTH* 99 (7): 1188–96. https://doi.org/10.2105/AJPH.2008.143131.

Carriedo, Angela, Adam D Koon, Luis Manuel Encarnación, Kelley Lee, Richard Smith, and Helen Walls. 2021. "The Political Economy of Sugar-Sweetened Beverage Taxation in Latin America:

Lessons from Mexico, Chile and Colombia." *Globalization and Health* 17 (1): 5. https://doi.org/10.1186/s12992-020-00656-2.

Cicchini, S., C. Russell, and K. Cullerton. 2022. "The Relationship between Volume of Newspaper Coverage and Policy Action for Nutrition Issues in Australia: A Content Analysis." Public Health 210: 8–15. https://doi.org/10.1016/j.puhe.2022.06.016.

Donaldson, Elisabeth A, Joanna E Cohen, Lainie Rutkow, Andrea C Villanti, Norma F Kanarek, and Colleen L Barry. 2015. "Public Support for a Sugar-Sweetened Beverage Tax and pro-Tax Messages in a Mid-Atlantic US State." *PUBLIC HEALTH NUTRITION* 18 (12): 2263–73. https://doi.org/10.1017/S1368980014002699.

Edsall Kromm, Elizabeth. 2008. "Framing Tobacco Taxes: Exploring the Construction of Print Media Coverage of Tobacco Excise Tax Initiatives in Six States and the Implications of Coverage for Media Advocacy." *Dissertation Abstracts International Section A: Humanities and Social Sciences*. ProQuest Information & Learning. https://search.ebscohost.com/login.aspx?direct=true&AuthType=ip,shib&db=psyh&AN=2008-99090-453&site=ehost-live&scope=site&authtype=ip,shib&custid=s3555202 NS -.

Elstein, Jeanette G., Caitlin M. Lowery, Puja Sangoi, Ana Peterhans, Sara N. Bleich, Hannah G. Lawman, and Christina A. Roberto. 2021. "Analysis of Public Testimony About Philadelphia's Sweetened Beverage Tax." *American Journal of Preventive Medicine* 000 (000): 1–10. https://doi.org/10.1016/j.amepre.2021.08.023.

Essman, Michael, Fernanda Mediano Stoltze, Francesca Dillman Carpentier, Elizabeth C Swart, and Lindsey Smith Taillie. 2021. "Examining the News Media Reaction to a National Sugary Beverage Tax in South Africa: A Quantitative Content Analysis." *BMC Public Health* 21 (1 PG-454): 454. https://doi.org/10.1186/s12889-021-10460-1.

Fogarty, Andrea S, and Simon Chapman. 2011. "Framing and the Marginalisation of Evidence in Media Reportage of Policy Debate about Alcopops, Australia 2008-2009: Implications for Advocacy." *DRUG AND ALCOHOL REVIEW* 30 (6): 569–76. https://doi.org/10.1111/j.1465-3362.2010.00253.x.

Fogarty, Andrea S, and Simon Chapman. 2013. "``Like Throwing a Bowling Ball at a Battle Ship" Audience Responses to Australian News Stories about Alcohol Pricing and Promotion Policies: A Qualitative Focus Group Study." *PLOS ONE* 8 (6). https://doi.org/10.1371/journal.pone.0065261.

Hagenaars, Luc L, Milica Jevdjevic, Patrick P T Jeurissen, and Niek S Klazinga. 2020. "Six Lessons from Introducing Sweetened Beverage Taxes in Berkeley, Cook County, and Philadelphia: A Case Study Comparison in Agenda Setting and Decision Making." *HEALTH POLICY* 124 (9): 932–42. https://doi.org/10.1016/j.healthpol.2020.06.002.

Hanim, Muhammad Faiz Mohd, Budi Aslinie Md Sabri, and Norashikin Yusof. 2021. "Online News Coverage of the Sugar-Sweetened Beverages Tax in Malaysia: Content Analysis." JMIR Public Health and Surveillance 7 (8). https://doi.org/10.2196/24523.

Hellman, Matilda, and Thomas Karlsson. 2012. "In Fear of a Reversal Back to the Spirits-Drinking Era - the 2004 Decrease of Finnish Alcohol Taxes in Public Discourse." *NORDIC STUDIES ON ALCOHOL AND DRUGS* 29 (1): 57–77. https://doi.org/10.2478/v10199-012-0005-z.

Hellowell, Mark, Katherine E Smith, and Alexandra Wright. 2016. "Hard to Avoid but Difficult to Sustain: Scotland's Innovative Health Tax on Large Retailers Selling Tobacco and Alcohol." *MILBANK QUARTERLY* 94 (4): 800–831. https://doi.org/10.1111/1468-0009.12200.

Hoe, Connie, Caitlin Weiger, and Joanna E Cohen. 2021. "The Battle to Increase Tobacco Taxes: Lessons from Philippines and Ukraine." *Social Science & Medicine* 279 (PG-114001): 114001. https://doi.org/https://doi.org/10.1016/j.socscimed.2021.114001.

Hua, Sophia V., Beth Uzwiak, Anastasia Hudgins, Ana Peterhans, Hannah G. Lawman, Sara N. Bleich, Jennifer Falbe, and Christina A. Roberto. 2022. "A Qualitative Study on Retailer Experiences with Philadelphia's Sweetened Beverage Tax." Translational Behavioral Medicine 12 (4): 554–67. https://doi.org/10.1093/tbm/ibab111.

James, Erin, Martin Lajous, and Michael R Reich. 2020. "The Politics of Taxes for Health: An Analysis of the Passage of the Sugar-Sweetened Beverage Tax in Mexico." *HEALTH SYSTEMS \& REFORM* 6 (1). https://doi.org/10.1080/23288604.2019.1669122.

Jun, Jungmi, Sei Hill Kim, James Thrasher, Yoo Jin Cho, and Yu Jin Heo. 2022. "Heated Debates on Regulations of Heated Tobacco Products in South Korea: The News Valence, Source and Framing of Relative Risk/Benefit." Tobacco Control 31 (e1): E57–63. https://doi.org/10.1136/tobaccocontrol-2020-056131.

Kane, Ryan M, and Vasanti S Malik. 2019. "Understanding Beverage Taxation: Perspective on the Philadelphia Beverage Tax's Novel Approach." *JOURNAL OF PUBLIC HEALTH RESEARCH* 8 (1): 40–45. https://doi.org/10.4081/jphr.2019.1466.

Marriott III, R W, and J P Dillard. 2020. "Sweet Talk for Voters: A Survey of Persuasive Messaging in Ten U. S. Sugar-Sweetened Beverage Tax Referendums." *Critical Public Health*, no. PG-. https://doi.org/10.1080/09581596.2020.1724263.

Moerschel, Katharina S., Peter von Philipsborn, Benjamin Hawkins, and Elizabeth McGill. 2022. "Concepts of Responsibility in the German Media Debate on Sugar Taxation: A Qualitative Framing Analysis." European Journal of Public Health 32 (2): 267–72. https://doi.org/https://doi.org/10.1093/eurpub/ckab200 Advance.

Niederdeppe, Jeff, Sarah E Gollust, Marian P Jarlenski, Ashley M Nathanson, and Colleen L Barry. 2013. "News Coverage of Sugar-Sweetened Beverage Taxes: Pro- and Antitax Arguments in Public Discourse." *AMERICAN JOURNAL OF PUBLIC HEALTH* 103 (6): E92–98. https://doi.org/10.2105/AJPH.2012.301023.

Park, C, and J Lee. 2020. "Stakeholder Framing, Communicative Interaction, and Policy Legitimacy: Anti-Smoking Policy in South Korea." *Policy Sciences* 53 (4 PG-637–665): 637–65. https://doi.org/10.1007/s11077-020-09394-z.

Purtle, Jonathan, Brent Langellier, and Felice Le-Scherban. 2018. "A Case Study of the Philadelphia Sugar-Sweetened Beverage Tax Policymaking Process: Implications for Policy Development and Advocacy." *JOURNAL OF PUBLIC HEALTH MANAGEMENT AND PRACTICE* 24 (1): 4–8. https://doi.org/10.1097/PHH.0000000000000563.

Raebeck, A, R Campbell, and E Balbach. 2010. "Unhealthy Partnerships: The Tobacco Industry and African American and Latino Labor Organizations." *J Immigr Minor Health* 12 (2): 228–33. https://doi.org/10.1007/s10903-009-9269-0.

Ramirez, Rebecca L, and David H Jernigan. 2017. "Increasing Alcohol Taxes: Analysis of Case Studies From Illinois, Maryland, and Massachusetts." *JOURNAL OF STUDIES ON ALCOHOL AND DRUGS* 78 (5): 763–70. https://doi.org/10.15288/jsad.2017.78.763.

Rao Seshadri, S, R Kaulgud, and P Jha. 2021. "'You Cannot Touch Taxes Easily': Making the Case for Tobacco Taxation in India." *Health Policy and Planning* 36 (3 PG-322–331): 322–31. https://doi.org/10.1093/heapol/czaa171.

Signal, Louise N, Carolyn Watts, Celia Murphy, Helen Eyles, and Cliona Ni Mhurchu. 2018. "Appetite for Health-Related Food Taxes: New Zealand Stakeholder Views." *HEALTH PROMOTION INTERNATIONAL* 33 (5): 791–800. https://doi.org/10.1093/heapro/dax019.

Watts, R A, K T Frick, and J Maddison. 2012. "Policy Making, Incrementalism, and News Discourse: Gasoline Tax Debates in Eight U.S. States." *Public Works Management and Policy* 17 (3 PG-238–255): 238–55. https://doi.org/10.1177/1087724X12437220.

Watts, Richard A, Sarah Heiss, Michael Moser, Jane Kolodinsky, and Rachel K Johnson. 2014. "Tobacco Taxes vs Soda Taxes: A Case Study of a Framing Debate in Vermont." *HEALTH BEHAVIOR AND POLICY REVIEW* 1 (3): 191–96. https://doi.org/10.14485/HBPR.1.3.3.

Zatoński, Mateusz Zygmunt, Catherine O Egbe, Lindsay Robertson, and Anna Gilmore. 2021. "Framing the Policy Debate over Tobacco Control Legislation and Tobacco Taxation in South Africa." *Tobacco Control* 0: 1–8. https://doi.org/10.1136/tobaccocontrol-2021-056675.

Zatonski, Mateusz, Benjamin Hawkins, and Martin McKee. 2018. "Framing the Policy Debate over Spirits Excise Tax in Poland." *HEALTH PROMOTION INTERNATIONAL* 33 (3): 515–24. https://doi.org/10.1093/heapro/daw093.

Zenone, Marco, and Nora Kenworthy. 2021. "Pre-Emption Strategies to Block Taxes on Sugar-Sweetened Beverages: A Framing Analysis of Facebook Advertising in Support of Washington State Initiative-1634." *Global Public Health* 0 (0): 1–14. https://doi.org/10.1080/17441692.2021.1977971.
