# Supplementary material for: Framing health taxes: a scoping review
Source: BMJ Glob Health. 2023 Oct 9;8(Suppl 8):e012055. doi: 10.1136/bmjgh-2023-012055 (PMC10565303; doi:10.1136/bmjgh-2023-012055)
Supplement: Supplementary data [file bmjgh-2023-012055supp003.pdf]

Year	Count	Percent
2022	4	10%
2021	13	33%
2020	5	13%
2019	1	3%
2018	4	10%
2017	1	2%
2016	1	2%
2014	2	5%
2013	2	5%
2012	2	5%
2011	1	2%
2010	1	2%
2009	1	2%
2008	1	2%
2006	1	2%
**Grand Total**	**40**	**100%**

Single Country?	Count	Percent
No	2	5.00%
Yes	38	95.00%
(blank)		0.00%
**Grand Total**	**40**	**100.00%**

Country	Count		
USA	17	17	43%
Australia	4	4	10%
Mexico	2	2	5%
United Kingdom	2	2	5%
South Korea	2	2	5%
South Africa	2	2	5%
New Zealand	1	1	3%
Ukraine	1	1	3%
Chile	1	1	3%
Poland	1	1	3%
Germany	1	1	3%

Admin Level	Count		
National	27	27	68%
City	9	9	23%
State	8	8	20%
County	3	3	8%
District	1	1	3%
Regional	1	1	3%
**Grand Total**	**49**		

Commodity	Count		
SSB	23	23	58%
Tobacco	12	12	30%
Alcohol	7	7	18%
Salt	1	1	3%
Ultra-processed foods	1	1	3%
Gas	1	1	3%
**Grand Total**	**45**		

Study Design	Count	Percent
Media/content analysis	20	50.00%
Case study	8	20.00%
comparative case study	5	12.50%
"qualitative"	5	12.50%
policy analysis	1	2.50%
focus group	1	2.50%
**Grand Total**	**40**	**100.00%**

Row Labels	Count	Percent

Data Source	Count		
News media	28	28	70%
Key Informant Interv	14	14	35%
gov docs	10	10	25%
industry docs	7	7	18%
NGO reports	5	5	13%
legislative proceedin	3	3	8%
Social Media	3	2	5%
radio	2	2	5%
television	1	1	3%
video	1	1	3%
mailers or billboards	1	1	3%
and conventional pri	1	1	3%
focus group discussi	1	1	3%
Survey	1	1	3%
documents	1	1	3%
**Grand Total**	**79**		

Theory	Count	Percent
Advocacy Coalition F	1	4.00%
Agenda-setting, fram	1	4.00%
e health policy triang	1	4.00%
Framing Theory	1	4.00%
Framing theory (com	1	4.00%
Framing theory (critic	2	8.00%
Framing theory (new	1	4.00%
Habermasian idea of	1	4.00%
Kingdon's 3 streams	2	8.00%
None	7	#####
Policy arenas and th	1	4.00%
Political Economy Ar	1	4.00%
Theory of framing wi	1	4.00%
(blank)		0.00%

1

Scotland	1	1	3%
Philippines	1	1	3%
India	1	1	3%
Colombia	1	1	3%
Ireland	1	1	3%
Malaysia	1	1	3%
Uganda	1	1	3%
Nigeria	1	1	3%
Finland	1	1	3%
**Grand Total**	**43**		

2020-2022	19	47.50%
2015-2019	10	25.00%
2010-2014	8	20.00%
2005-2009	3	7.50%
**Grand Total**	**40**	**100.00%**

Framing theory (Gam	1	4.00%
Framing Theory (critic	1	4.00%
agenda-setting (light	1	4.00%
Framing (communica	1	4.00%
**Grand Total**	**25**	**#####**

Row Labels	Count	Percent
Health Promotion International	3	7.50%
PLoS One	2	5.00%
Social Science & Medicine	2	5.00%
Public Health Nutrition	2	5.00%
American Journal of Public Health	2	5.00%
Tobacco Control	2	5.00%
Health Policy and Planning	2	5.00%
Dissertation	1	2.50%
Journal of Public Health Research	1	2.50%
Journal of Immigrant and Minority Health	1	2.50%
Global Public Health	1	2.50%
Critical Public Health	1	2.50%
Globalization and Health	1	2.50%
Drug and Alcohol Review	1	2.50%
Health Behavior & Policy Review	1	2.50%
Journal of Public Health Management and	1	2.50%
Health Policy	1	2.50%
Journal of Studies on Alcohol and Drugs	1	2.50%
The Milbank Quarterly	1	2.50%
Policy Sciences	1	2.50%
BMC Public Health	1	2.50%
Public Works and Management Policy	1	2.50%
American Journal of Preventive Medicine	1	2.50%
Global Health Action	1	2.50%
Translational Behavioral Medicine	1	2.50%
Versita	1	2.50%
JMIR Public Health and Surveillance	1	2.50%
European Journal of Public Health	1	2.50%
Public Health	1	2.50%
Health Systems & Reform	1	2.50%
American Journal of Health Promotion	1	2.50%
Journal of Epidemioly and Community Hea	1	2.50%
**Grand Total**	**40**	**100.00%**

Koon AD, Marten R. *BMJ Glob Health* 2023; 8:e012055. doi: 10.1136/bmjgh-2023-012055

Organization Type Count				
NGO	32.00	32.00	80%	**Avg**
industry associate	30.00	30.00	75%	6.00
corporate entity	27.00	27.00	68%	
ministry/dept of he	26.00	26.00	65%	
legislative politiciar	21.00	21.00	53%	
media	20.00	20.00	50%	
university	16.00	16.00	40%	
executive branch p	15.00	15.00	38%	
treasury/ministry of	15.00	15.00	38%	
multilateral	10.00	10.00	25%	
professional health	8.00	8.00	20%	
Private philanthrop	5.00	5.00	13%	
judicial politician	4.00	4.00	10%	
regulatory agency	1.00	1.00	3%	
Chamber of Comm	1.00	1.00	3%	
medical experts	1.00	1.00	3%	
City officials (gener	1.00	1.00	3%	
public relations firm	1.00	1.00	3%	
social influencer/ce	1.00	1.00	3%	
economists private	1.00	1.00	3%	
residents	1.00	1.00	3%	
advocacy groups	1.00	1.00	3%	
retailers	1.00	1.00	3%	
think tanks	1.00	1.00	3%	
**Grand Total**	**240.00**			

Pro-tax MF	Count		
Care/harm	35	35	88%
fairness/cheating	18	18	45%
liberty/oppression	17	17	43%
sanctity/degredatic	10	10	25%
authority/subversic	7	7	18%
loyalty/betrayal	5	5	13%
**Grand Total**	**92**		

Anti-tax MF	Count		
fairness/cheating	26	26	65%
liberty/oppression	21	21	53%

Tax Type	Count		
excise	30	30.00	75%
value added/wholesale/gross reciepts	7	7.00	18%
retail transaction	2	2.00	5%
import duty	1	1.00	3%
vendor priviledge	1	1.00	3%
**Grand Total**	**41**		

Outcome	Count		
Old tax modified	14	14	35%
New tax created	14	14	35%
Contestation (unresolved)	12	12	30%
New tax rejected	5	5	13%
New tax modified	2	2	5%
(blank)			
**Grand Total**	**47**		

CPA	Count		
shaping the evidence -> Lobbying	29	29.00	73%
shaping the evidence -> shaping research	8	8.00	20%
constituency building -> promoting or spor	8	8.00	20%
constituency building -> financial or non-fir	7	7.00	18%
contributing to health policy consultations	6	6.00	15%
partnership of voluntary agreements with (	5	5.00	13%
(blank)			
**Grand Total**	**63**		

Misc	Count		
Jingle or slogan	11	11	28%
series of events	9	9	23%
social media influencers	1	1	3%
wearables	1	1	3%
celebrity endorsement	1	1	3%
petitions	1	1	3%
**Grand Total**	**24**		
			0%

Care/harm	18	18	45%
authority/subversic	10	10	25%
loyalty/betrayal	8	8	20%
sanctity/degredatic	4	4	10%
**Grand Total**	**87**		

**Pro-tax Value**	**Count**		
welfare	34	34	85%
Equity	21	21	53%
Efficiency	21	21	53%
liberty	16	16	40%
security	11	11	28%
**Grand Total**	**103**		

**Anti-tax Value**	**Count**		
Efficiency	21	21	53%
liberty	21	21	53%
Equity	19	19	48%
welfare	19	19	48%
security	12	12	30%
**Grand Total**	**92**		

**Frame Type**	**Count**		
Policy action (surface level)	30	30	75%
Institutional (intermediate)	10	10	25%
Metacultural (deep/broad)	4	4	10%
(blank)			
**Grand Total**	**44**		

**Argument For**	**Count**		
Reduces suffering and premature death	33	33	83%
lucrative for governments	22	22	55%
cost containment/savings	19	19	48%
pro-poor policy	14	14	35%
education	9	9	23%
everyone else is doing it	9	9	23%
product reformation	5	5	13%
cheap	4	4	10%
**Grand Total**	**115**		

**Argument Against**	**Count**		
threat to industry	25	25	63%
tax on the poor	23	23	58%
hurts/eliminates jobs	21	21	53%
better means to end	21	21	53%
narrow and unfair	19	19	48%
meaningless (too small/ineffective)	18	18	45%
nanny state	17	17	43%
Promote illicit trade	5	5	13%
**Grand Total**	**149**		

Koon AD, Marten R. *BMJ Glob Health* 2023; 8:e012055. doi: 10.1136/bmjgh-2023-012055
